# Regional radiotherapy after primary systemic treatment for cN+ breast cancer patients

**DOI:** 10.1016/j.breast.2023.02.006

**Published:** 2023-02-15

**Authors:** Liesbeth J. Boersma, Ingvil Mjaaland, Frederieke van Duijnhoven

**Affiliations:** a Dept. Radiation Oncology (Maastro), GROW-School for Oncology and Reproduction, Maastricht University Medical Centre+, Maastricht, the Netherlands; b Division of Medicine, Stavanger University Hospital, Stavanger, Norway; c Dept. Surgery, The Netherlands Cancer Institute, Amsterdam, the Netherlands

**Keywords:** Radiation oncology, Regional nodal irradiation, Axillary management, Primary systemic treatment, de-escalation, ALND, Axillary Lymph Node Dissection, ART, Axillary Radiation Therapy, IMN, Internal Mammary Nodes, LRR, Loco Regional Recurrence risk, MARI, Marking of the Axilla with Radioactive Iodine, PMRT, Post Mastectomy Radiation Therapy, PST, Primary Systemic Therapy, RNI, Regional Nodal Irradiation, RT, Radiation Therapy, SARP, Surgical Axillary Restaging Procedures, SLNB, Sentinel Lymph Node Biopsy

## Abstract

Pathologic complete response (pCR) after Primary Systemic Treatment (PST) for breast cancer is associated with excellent long-term outcomes. With increasing use of PST, the indication for regional nodal irradiation (RNI) has been challenged. The aim of this paper is to review the literature on de-escalation of RNI in patients treated with PST.

We found no level 1 evidence on de-escalation of RNI after PST, but several randomized trials are ongoing. Consequently, current de-escalation strategies are based on cohort studies. These studies showed that in patients with *low* nodal tumour burden (LNTB) (≤3 suspicious nodes at imaging) prior to PST, and ypN0 based on Axillary Lymph Node Dissection (ALND), omission of RNI resulted in very low regional recurrences (RR) without compromising survival. In patients with LNTB and ypN0 based on Sentinel Lymph Node Biopsy (SLNB), omission of axillary treatment also resulted in low RR; the majority of these patients received local radiotherapy. Similarly, in patients with ypN1 (ALND) disease, omission of RNI resulted in low 5-year RR rates. Low RR-rates were also found in the few studies replacing ALND by RNI, in patients with ypN1 (SLNB) disease.

In patients with *high* nodal tumour burden prior to PST and ypN0 (SLNB), replacing ALND by RNI also resulted in low RR. Due to the limited number of patients, these data should be interpreted with caution.

We conclude that although level 1 evidence is lacking, de-escalation of RNI after PST can be considered in selected cases.

## Highlights

• Regional Nodal Irradiation (RNI) is not always indicated after PST in cN + patients.
• There is no level 1 evidence on de-escalation of RNI after PST, RCTs are ongoing.
• Data from cohorts suggest that in specific situations RNI can replace ALND.
• Data from cohorts suggest that in specific situations RNI can be omitted after PST.
• Further studies on de-escalating RNI are required, considering tumour biology.

## 1 Introduction

Nowadays, an increasing number of breast cancer patients is being treated with primary systemic treatment (PST), instead of primary surgery [1]. Patients are eligible for PST when based on the diagnostic examinations, it is already clear that systemic treatment is indicated. A disadvantage of PST may be that the indications for radiation therapy (RT) are less clear, since they are traditionally based on the pathological features obtained at primary surgery [2]. PST however has important advantages compared to adjuvant systemic treatment: 1) the response can be monitored, which can be motivating for the patient to continue systemic treatment, and it can be used to adapt systemic treatment; 2) the patient is allowed more time to think about the surgical options, for which sometimes genetic analysis may be required; 3) downstaging of the tumour yields a higher chance of safe breast conserving surgery [3]; and 4) downstaging of axillary nodes may allow de-escalation of the nodal treatment [[4], [5], [6], [7], [8], [9], [10], [11], [12]]. Especially the latter two aspects are gaining interest, since long term quality of life of survivors is becoming increasingly important with the impressive increase in survival rates of breast cancer. Consequently, several studies are now focusing on de-escalation of treatment based on the response to PST, aiming to improve quality of life, without compromising overall survival.

Both axillary lymph node dissection (ALND) and nodal irradiation (i.e. RT of level 1–4 [13]) can have detrimental effects on quality of life by causing lymphoedema, and/or impaired shoulder function [14]. Therefore, in the setting of primary surgery, following the introduction of SLNB several decades ago, ALND has been limited to patients with clinically positive nodes, or with a positive sentinel lymph node biopsy (SLNB), thereby accepting a <5% false negative rate for the SLNB [[15], [16], [17], [18]]. Subsequently, the AMAROS and OTOASOR trials [14,19,20] showed that, in the setting of primary surgery and a positive SLNB, replacing ALND by Axillary RT (ART) yielded oncologically similar results, whilst reducing the risk of lymphoedema of the arm. The ACOSOG Z0011 trial and IBCSG 23–01 trials [[21], [22], [23]], performed in the setting of primary surgery and a positive SLNB, even showed that omission of any further axillary treatment seems to be oncologically safe, in a group of patients of whom the far majority also underwent whole breast RT and adjuvant systemic treatment. More recent trials have gone a step further, by omitting SLNB in patients with cT1-2cN0 disease [24,25]. Results of these trials are still pending. Whilst axillary surgery is being de-escalated due to a lack of survival benefit, regional nodal radiotherapy (RNI, i.e. RT of axillary (Level 1&2), periclavicular (Level 3&4), and/or internal mammary nodes (IMN)), has been shown to improve overall survival in patients with positive nodes in the setting of primary surgery, especially in case of >3 involved nodes [26,27]. Consequentially, an increase in the application of RNI is seen. One of the problems when interpreting the literature however, is that often the extent of RNI is not explained. In this paper we will use the term RNI when no additional information is given: in most cases this includes at least Level 3 and 4. Where possible we will add more detail.

In summary, in the setting of primary surgery, regional treatment is increasingly being individualized, from 1) no SLNB and no axillary treatment at all in low risk patients, to 2) omitting ALND or replacing ALND by ART in cN0/pN+ (SLNB) patients, and to 3) ALND in combination with RT of Level 3 & 4 nodes with or without inclusion of IMN, especially in patients with pN2 disease [[26], [27], [28], [29]].

Similar developments are ongoing after PST, as studies are being performed to investigate whether regional nodal treatment can be de-escalated based on response to PST. Since it has been shown that an axillary pCR can be reached in patients with initially involved nodes in 44 up to even 97% in ER negative Her2 positive tumours [[30], [31], [32], [33], [34]], there is an increasing demand for de-escalation of axillary treatment.

The aim of this paper is to review the literature on de-escalation of RNI in patients treated with PST. We will first discuss RNI after ALND, and secondly focus on de-escalation of axillary treatment.

## 2 De-escalation of regional nodal radiotherapy after PST and ALND

As mentioned above, RNI after ALND in the setting of primary surgery has been shown to yield a survival benefit, especially in case of 4 or more involved nodes [[26], [27], [28], [29]]. With the advent of PST, several studies have analysed the oncological outcome in patients who underwent ALND following PST. The MD Anderson group published one of the earliest series of patients treated with PST in relatively early breast cancer [2,[35], [36], [37]]. It was concluded that the locoregional recurrence risk (LRR) was dependent on both the pre-and post PST tumour stage and tumour biology. In 2012, Mamounas [38] performed a combined analysis of the NSABP B-18 and B-27 trials, in which patients were treated with PST followed by breast conserving surgery or mastectomy including ALND. RT was limited to the breast in case of breast conserving surgery; no RNI was given. This analysis showed that ypT and ypN-status were independent predictors for LRR both after breast conserving therapy and mastectomy [38]. In a more recent analysis [39], data of the NSABP B-40 and B-41 trials were included in the analysis as well. In these trials patients were treated with PST, breast surgery and ALND, and RNI was left to the discretion of the physician. In univariate analysis, only patients with ypN + Her2+ disease who received RNI had improved overall survival when compared to non-irradiated patients; whereas improved LRR was seen in B-40 patients with ypN + hormone receptor positive disease. However, in the multivariable analysis of the B-40 and B-41 study populations, RNI was not found to be significantly associated with improved OS, disease-free survival, distant recurrence, or LRR.

Several cohort studies have been published analysing the impact of postmastectomy RT (PMRT) on LRR and overall survival after PST and ALND. Unfortunately, most of these studies did not specify whether and/or which regional nodal volumes were included in the PMRT. A meta-analysis by Wang in 2021 [40] included 12 studies published between 2004 and 2019 containing data of 17.747 patients treated with PST. They found that PMRT was associated with a lower LRR (RR 0.38; 95% CI, 0.19–0.77, P = 0.007), especially in patients with stage III breast cancer (RR 0.16; 95% CI, 0.07–0.37, P < 0.001). However, PMRT did neither result in improved disease-free survival in ypN0 patients (RR 0.70; 95% CI, 0.21–2.27, P = 0.55), nor in a better overall survival (RR 0.81; 95% CI, 0.64–1.04, P = 0.10).

Wang et al. [41] developed a nomogram to predict the 3 and 5 -year LRR, including ypN-status, lymph angioinvasion, and histological grade, based on 217 cT1-2N0-1 patients, who were treated with PST, mastectomy and ALND; 128 patients received PMRT, i.e. 25 × 2 Gy to the chest wall, regional nodal basins (high axilla and supraclavicular fossa, with or without the internal mammary chain), and 89 patients did not receive PMRT. Only patients with a high risk of a LRR based on this nomogram benefitted from PMRT. In two cohort studies that also included patients with breast conserving therapy, more detailed data on RT after PST and ALND were available. First, Haffty et al. [42] analysed the data of the ACOSOG 1071 trial (Alliance), which included patients with stage I-III disease. They found that locoregional RT did lower the 5-yr LRR rate in patients with ypN + disease, but not in patients with ypN0 disease. Recently, De Wild et al. [43] presented the data of the RAPCHEM study; in this prospective cohort study, locoregional RT was de-escalated according to predefined study guidelines based on the ypN status in patients with cT1-2N1 disease, with ≤3 suspicious nodes at imaging. Six hundred eighty-one of the included 838 patients underwent ALND. Based on the predefined guidelines, only whole breast RT was recommended after breast conserving surgery in patients with ypN0 disease; after mastectomy no RT was delivered at all (N = 291). After 5 years the overall LRR in this group was 2.2%; in the patients where the study guidelines (N = 181) were actually followed, it was 2.3%. In patients with ypN1 status, only local RT without RNI was recommended, both after breast conserving surgery and mastectomy (N = 370). The 5-year LRR risk in this group was also very low at 2.2%, and in patients in whom the study guidelines were actually followed (N = 200) it was even lower at 1%. In both the study of Haffty et al. [42] and De Wild et al. [43], triple negative disease was an independent predictor for LRR, even in the ypN0 groups.

In an analysis of three German trials [44] however, patients with ypN0 disease still had a high 5-yr LRR of 15.2%. This was probably caused by the fact that more high-risk patients (45% stage III) were included. The results of the ongoing NSAPB-51/RTOG 1304 (NCT01872975) study will shed further light on the effect of RNI after PST. In this trial patients with cT1-3N1 disease are treated with PST, followed by breast surgery and ALND or SLNB only. In case of axillary pCR, patients are randomized to RNI (including the IMN), or no RNI. Patients treated with a mastectomy do not receive any RT at all if randomized to no RNI. Although we are eagerly awaiting the results, Vega [39] expressed concerns that the results may be biased since there is no stratification on breast residual disease, which was found to be an important predictor in other analyses [38,39,42], especially in patients with triple negative disease.

*In summary,* the above-mentioned studies strongly suggest that RNI can be omitted in patients with cT1-2N1 (and ≤3 or less involved nodes), ypN0 (ALND) disease, possibly with the exception of very poor tumour biology (e.g. triple negative, no breast pCR) or very young age. In patients with stage III disease or higher however, there is still general consensus that these will benefit from post-operative RT, regardless of their response to PST [45,46].

## 3 De-escalation of axillary treatment

In this section, we will first shortly review studies aimed at restaging the axilla as a pre-requisite to select patients for de-escalation of axillary treatment. Subsequently, studies investigating de-escalation of axillary treatment will be reviewed for both ypN0 and ypN1 patients.

### 3.1 Restaging of the axilla

Several studies have investigated the use of non-invasive methods to re-stage the axilla after PST, in patients with involved nodes prior to PST. Both Ng et al. [47] and Samiei et al. [48] showed that the biologic subtype is highly correlated with ypN status, with the highest axillary pCR rates in hormone receptor negative and Her2-receptor positive tumours (60–78%), and the lowest nodal pCR rate in luminal A breast cancer (13%). Schipper et al. [49] found in a review of non-invasive response assessment methods that clinical examination, axillary ultrasound, MRI, FDG-PET-CT and a nomogram were not sufficiently accurate to predict an axillary pCR, and thus to decide on the omission of any axillary treatment.

This was confirmed in a more recent review of Singer et al. [50], who additionally reviewed the value of SLNB after PST both in cN0 and in cN + disease. They concluded that in patients with cN0 disease, SLNB is widely accepted as a staging procedure after PST, since the false negative rate is comparable to the false negative rate (FNR) of SLNB in primary surgery. In patients with cN + disease, several studies have been performed to investigate the value of SLNB after PST; the false negative rates observed in the GANEA1, ACOSOG Z1071, SN FNAC, SENTINA and trials ranged from 8.4% to 14.2% [34,[51], [52], [53]] and was related to the number of removed nodes, the mapping agents (single or dual tracer) and the pathological analysis of the node (using immune histochemistry or not).

Other studies investigated the use of different techniques to mark pathologic lymph nodes before start of PST, allowing selective removal of these nodes after PST. Donker et al. [54] reported on 100 patients in whom the MARI procedure was performed, i.e. where the node was marked with a radio-active iodine seed. The false negative rate in this study was 7%. Others combined SLNB with removal of a clipped node, i.e. Targeted Axillary Dissection (TAD), and found false negative rates of 2% and 3.5%, respectively [55,56]. Nevertheless, it can be questioned whether removal of a marked node eventually is really required, since there is an increasing number of reports that suggests that regional recurrences are rare if ALND is omitted in patients with ypN0 (SLNB) disease (see also next section). Nowadays, all types of surgical axillary re-staging procedures (SARPs) are being applied in clinical practice.

### 3.2 De-escalation of axillary treatment after PST in case of clinically positive, ypN0 (SARP)

De-escalation of axillary treatment can be reached by replacing ALND with axillary RT (ART) or by omitting axillary treatment altogether, i.e. by omitting ALND without adding ART. Several studies have been performed and are currently ongoing, to tailor the degree of de-escalation to the individual patient, based on the results of the surgical axillary restaging procedure (SARP).

#### 3.2.1 De-escalation of axillary treatment in case of ypN0 (SARP)

##### 3.2.1.1 Outcome data of single arm cohort studies

As a first de-escalation step, some studies analysed whether it was safe *to replace ALND with ART* in case of ypN0 (SARP). In the prospective cohort study NEOSENTI-TURK MF-18-02 [57], 211 patients with cN+, ypN0 (SLNB) were treated with RNI (axilla level 1–4) instead of ALND. Only one patient developed a nodal recurrence 60 months after treatment. They found that patients with cT3/4, non-luminal molecular pathology had a significantly lower 5-year DFS than cT1/2 patients. They did not find a difference in 5-year DFS between patients with ypN0 (SLNB) disease and ypN1mi (SLNB), in whom ALND was omitted as well.

In 2018, van der Noordaa et al. [58] described the Dutch de-escalation strategy for axillary treatment based on the axillary status according to the nodal tumour burden on PET-CT prior to PST, in combination with the ypN status based on the MARI procedure. In patients with high nodal tumour burden, i.e. > 3 suspicious axillary nodes on PET-CT, and axillary complete response (ypN0 (MARI)), ALND was replaced with RT of Level 1–4. With 3 years of follow-up, none of the 98 patients developed an axillary recurrence [12].

In the last two years, an increasing number of reports has been published on even further de-escalation in case of ypN0 (SARP): *omission of any axillary treatment, i.e. omission of ALND without specifically adding ART* [[4], [5], [6], [7], [8], [9], [10], [11], [12],^43^]. All studies were retrospective and included heterogeneous populations; the studies used different inclusion criteria varying from cN0 to cN3 disease, and follow-up varied from 3 to 10 years. In these studies, ALND was omitted in case of ypN0 (SARP) disease, but only a limited number of studies presented details on administration of RT; especially data on RT to the breast or thoracic wall were largely lacking. Most report that patients treated with breast conserving therapy received whole breast RT, thereby probably including the lower axilla, and/or they reported which percentage of patients received RT, but it is difficult to assess in which patients no axillary treatment at all was applied. The number of cN+/ypN0 (SARP) patients in whom regional treatment was omitted varied, from 38 [10] to 125 [4]. All studies reported a low axillary recurrence rate of <5%.

In two Dutch studies, more detailed information on RT was available. In the above described Dutch strategy [58], it was proposed to omit any axillary treatment in case of low nodal tumour burden (≤3 involved axillary nodes on PET-CT) prior to PST, and ypN0 (MARI) status. After a follow-up of 3 years, axillary recurrence developed in one out of 56 patients [12]. The same strategy was also followed in the RAPCHEM study, where axillary re-staging was performed either by the MARI or SLNB procedure in a very limited subgroup [43]. In 41 patients with cT1-2N1, ypT0-2ypN0(SARP) breast cancer, any axillary treatment was omitted and only 1 patient had a locoregional recurrence after 5-year follow-up.

To investigate the possible detrimental effect on overall survival of omitting RNI in case of cN+, ypN0 (SLNB), Schlafstein et al. [8] used the National Cancer Database, to analyse 10-year survival of 1963 patients who were treated with PST, followed by lumpectomy and SLNB, between 2006 and 2015. The application of RNI increased from 48% to 62%. However, adding RNI to whole breast irradiation (WBI) did not improve 10-year survival: for WBI 10-year OS was 83.6%, vs 79.5% for WBI plus RNI. In multivariate analyses, adding RNI was not associated with a survival benefit. The authors recognized that this retrospective study may have been subject to several biases such as absence of data on exact radiation fields, number of removed SLNs and pathology of nodal status prior to PST. Consequently, they advise to await further studies before definitive conclusions can be drawn.

##### 3.2.1.2 Ongoing studies in ypN0 (SARP) disease

Several studies exploring the issue of de-escalating axillary treatment after PST are still ongoing. As a follow-up of the RAPCHEM study [43], the Minimax study is a prospective cohort study in The Netherlands evaluating the Dutch strategy to de-escalate axillary treatment; this study will report on the outcome of data accrued between 2015 and 2021 [59]; in another prospective cohort study, the Eubreast Axsana (NCT04373655) study [60] different SARPs and outcomes of patients treated between 2020 and 2030 are prospectively recorded.

Several randomized trials are ongoing as well, investigating de-escalation of axillary treatment in case of cN1/ypN0 (Table 1). In the randomized NSABP-51 trial/RTOG 1304 (NCT01972975) patients with cT1-3N1 breast cancer, treated with PST resulting in ycN0 disease, undergo breast surgery with SLNB or ALND. Patients with a nodal pCR, ypN0 (SLNB) or ypN0 (ALND), are randomized between no additional RT (or only breast RT), and RT of level 1–4, including the IMN. This study aims to include 1636 patients between 2013 and 2023. The primary endpoint is 10-year Disease-Free Survival.

**Table 1:** Ongoing RCTs on de-escalating axillary treatment in patients with ypN0 disease. SLNB: Sentinel Lymph Node Biopsy; ALND: Axillary Lymph Node Dissection; (A)RT: (Axillary) Radiation Therapy, i.e. level 1 and 2; DFS: Disease-Free- Survival.

	Inclusion criteria	Randomization arms	Inclusion period and number of patients to include	Primary endpoint
**NSABP-51/RTOG 1304 NCT01972975**	cT1-3N1, ycN0, undergoing breast surgery and ypN0 (SLNB or ALND)	No additional RT (only breast RT in case of breast conservation) vs Regional Nodal RT, i.e. Level 1–4 and IMN	2013–2023	10-year DFS
			N = 1636	
**ATNEC NCT 0410979**	cT1-3N1, ycN0, undergoing breast surgery and ypN0(TAD)	No axillary treatment (No ART, and no ALND) vs Axillary treatment (ART or ALND)	2021–2030	5-year DFS, and 5 year Lymph-oedema of the arm
			N = 1900	

The inclusion criteria for the randomized ATNEC trial (NCT 0410979) are similar to the NSABP-51/RTOG 1304, but the axillary staging after PST consists of TAD rather than SLNB/ALND. In case of ypN0(TAD), patients are randomized between no axillary treatment at all (no ART, no ALND), or axillary treatment (either ALND or ART). This study aims to include 1900 patients between 2021 and 2030. The primary endpoints are 5-year Disease-Free Survival and 5-year incidence of arm lymph-oedema.

*In summary,* up till now the retrospective heterogeneous data that are available in patients with cN0/N1-2/ypN0 (SARP) disease suggest that omitting further axillary treatment in these patients is oncologically safe (Table 3). However, in the above described studies, there is still considerable uncertainty about the impact of (incidental) radiation dose to the axilla, or dose to the axilla not allowed according to the protocol [61], on the recurrence rate. Further, there may be considerable variation between studies in the cN status of the patients, since it is often not clear 1) whether and which axillary imaging was performed to determine the clinical cN status, and 2) how the clinical N status was defined, which in part is caused by a discrepancy between cN2 and pN2 definition in the official TNM definition (UICC): in the official TNM definition, cN2 disease comprises metastasis in ipsilateral level 1 and 2 of the axillary lymph node(s) that are clinically fixed or matted or in clinically detected ipsilateral internal mammary lymph node(s) in the absence of clinically evident axillary lymph node metastasis, whereas pN2 disease is defined as ≥ 4 involved nodes. We cannot exclude that in many papers, the investigators have defined cN1 disease as ≤ 3 suspicious nodes, as would be the case in pN1 disease, and classified patients as cN2 disease in case of ≥4 suspicious nodes at imaging. Hence, we would recommend to only consider omission of axillary treatment in patients with ypN0 (SARP) disease, who had ≤3 suspicious nodes prior to PST (Table 3), who are at least irradiated to the breast or chest wall, thereby taking into account tumour biology (e.g. triple negative disease, pCR), stage prior to PST [35] and age. The results of further studies are to be awaited before definitive conclusions can be drawn. If one considers not to await these results, de-escalation should be performed in prospective cohort studies.

**Table 2:** Ongoing RCTs on de-escalating axillary treatment in patients with ypN1 disease. SLNB: Sentinel Lymph Node Biopsy; ALND: Axillary Lymph Node Dissection; (A)RT: (Axillary) Radiation Therapy, i.e. level 1 and 2; DFS: Disease-Free- Survival.

	Inclusion criteria	Randomization arms	Inclusion period and number of patients to include	Primary endpoint
**ALLIANCE 11202 NCT 01901094**	cT1-3N1, ypN1(SLNB)	ART vs ALND	2014–2024	5-year DFS
		All patients receive RT to level 3 and 4 and IMN	N = 2918	
**ADARNAT NCT04889924**	cT1-T4bN0-1, ycN1 (<4 involved nodes); ypN1 (SLNB with ≤2 macrometastases)	ART vs ALND	2021–2026	5-year DFS
		All patients receive RT to level 3 and 4 and IMN	N = 1666	
**TAXIS NCT 03513614**	cN1-2, ypN+ and removal of all clinically suspicious nodes	ALND and locoregional RT excluding the dissected axilla vs. Locoregional RT *including the axilla*	2018–2029	20-year DFS
			N = 1500

**Table 3:** Conclusions from literature on de-escalating axillary treatment. SARP: Surgical Axillary Staging Procedure, i.e. sentinel lymph node biopsy (SLNB), removal of marked node(s), or both; ART: axillary radiotherapy; ALND: Axillary Lymph Node Dissection; RCT randomized controlled clinical trials.

	ypN0 (SARP): Omit further axillary treatment?	ypN1 (SARP): Replace ALND with ART?	ypN2 (SARP)
**cN0**	Yes, most probably safe. To be confirmed in prospective cohort studies.	Consider ART instead of ALND, probably safe; preferably apply within at least a registration study.	ALND followed by RT of the breast/chest wall in combination with Level 3 and 4 and the undissected axilla, plus or minus the IMN. TAXIS RCT to be awaited to study replacing ALND by ART.
		Cohort studies to be awaited.	
**cN1 – Low Nodal Tumour Burden (≤3 suspicious nodes at imaging)**	Consider to omit, probably safe, but preferably apply within at least a registration study.	Consider ART instead of ALND, probably safe; preferably apply within at least a registration study.	
	Cohort studies and NSABP-51 and ATNEC -RCTs to be awaited.	Cohort studies and Alliance 11202, ADARNAT, TAXIS - RCTs to be awaited.	
**cN1 – High Nodal Tumour Burden (> 3 suspicious nodes at imaging)**	Do not (yet) omit, more data required. NSABP-51 and ATNEC -RCTs to be awaited.	Be reluctant; if ART is considered instead of ALND, only within at least a registration study. Cohort studies and Alliance 11202, ADARNAT, TAXIS - RCTs to be awaited.	

#### 3.2.2 De-escalation of axillary treatment in case of ypN+ (SARP)

##### 3.2.2.1 Outcome data of single arm cohort-studies

Only very limited data are available on de-escalating axillary treatment in patients with ypN+ (ALND), and most studies de-escalate treatment by replacing ALND with RT. Although the AMAROS and OTAOSOR trials [14,20], ACOSOG Z0011 [21,22] and IBCSCG 23–01 [23] studies suggested that in patients with pN1 (SLNB) disease, replacing ALND by ART or even omitting any further axillary treatment was oncologically safe, these studies were performed in the setting of primary surgery, where the vast majority of patients received adjuvant systemic treatment. In addition, as mentioned earlier, the results of the ACOSOG Z0011 study may be influenced by incidental radiation dose to the axilla by a high tangential field or directed nodal irradiation via a third field, contrary to protocol requirements [61]. Consequently, the results of these trials cannot be translated into the setting of PST without further studies, and therapeutic recommendations based on these studies must be made with extreme caution.

Nevertheless, replacing ALND by ART is being investigated, based on extrapolating the results of the AMAROS trial to the PST setting, assuming that 1) the AMAROS trial showed that microscopic disease in the axilla can be eradicated by ART; and 2) most patients treated with PST receive adjuvant systemic treatment as well: hormone receptor positive patients always receive adjuvant endocrine treatment, Her-2 positive patients receive adjuvant targeted treatment, and triple negative patients receive adjuvant capecitabine in case of a non pCR [62]. Consequently, the only patients not receiving adjuvant systemic treatment after PST are patients with triple negative disease and pCR.

In the above described Dutch approach, ALND was replaced by RT to level 1–4 in 118 patients with low nodal tumour burden prior to PST (i.e. ≤3 axillary nodes on PET-CT), and a positive MARI node after PST [12]; at 3 years median follow-up, 4 patients had an axillary recurrence, all synchronous with other recurrent localisations (one local, one regional and two distant), all of whom had triple negative disease. In a similar subgroup of the RAPCHEM study, none out of 24 patients had an LRR after 5 years follow-up [43].

Although these limited data may suggest that replacing an ALND by ART is safe in patients with low nodal tumour burden and ypN1 (SLNB/MARI) disease, Almahariq et al. [63] found that replacing ALND with RNI in patients with cT1-3N1 disease, ypN1(SLNB) was associated with a significant lower 5-year survival (71%) than performing an ALND plus RNI (77%). Only in patients with luminal A or B tumours with a single metastatic lymph node, 5-year overall survival was similar (exploratory subgroup analyses). Their analysis was based upon 1617 breast cancer patients in the national cancer database, treated between 2004 and 2014, and due to lack of information on SLNB or ALND, they defined SLNB as removal of ≤4 nodes, and ALND as removal of >4 nodes. Eighty-one percent underwent ALND followed by RNI, and 19% underwent SLNB followed by RNI. The two groups were matched for patient, tumour, and treatment characteristics. They found that after publication of the ACOSOG Z0011, ALND was omitted even more often, i.e. in 30% in 2014. In this study, no data were available on the number of involved nodes prior to PST.

##### 3.2.2.2 Ongoing studies in ypN+ (SLNB) disease

In a follow-up of the RAPCHEM study, the Minimax study is ongoing, where the outcome of the Dutch strategy will be analysed in patients treated between 2015 and 2021 [59]. As mentioned above (paragraph on ypN0(SARP)), the prospective cohort study Eubreast Axsana (NCT04373655) will report as well on the different axillary restaging approaches and the outcomes of patients treated between 2020 and 2030, also in case of ypN+ [60].

In addition, several randomized trials are investigating whether ALND can be replaced by RT in case of ypN + disease (Table 2). The ALLIANCE trial 11202 (NCT 01901094) randomizes patients with cT1-3N1/ypN1(SLNB) disease between ART level 1 and 2 and ALND. All patients are treated with RT to level 3 and 4 and the IMN. This study aims to include 2918 patients between 2014 and 2024. The primary endpoint is 5-year Disease Free Survival. In 2021, the ADARNAT trial (NCT04889924) opened; this trial closely resembles the ALLIANCE 11202, but in addition includes patients with T4b disease. It aims to include 1666 patients between 2021 and 2026, and also has 5-year Disease Free Survival as primary endpoint. The TAXIS trial (NCT 03513614) [64] opened in 2018; in this trial, node positive patients treated with primary surgery or with PST are included. Breast surgery is combined with removal of all clinically suspicious nodes. Subsequently, (y)pN + patients are randomized to 1) completion ALND and locoregional RT, or to 2) locoregional RT *including the axilla.* This study aims to accrue 1500 patients between 2018 and 2029, and has as primary endpoint 20-year Disease Free Survival.

The Neonod2 study (NCT04019678) is an Italian prospective cohort study for patients where axillary treatment (no ALND nor ART) is omitted completely in patients with cT1-3N+, ypN1mi (SLNB) disease. This approach is similar to the approach in the IBCSG 23-01 study [23] which was performed in patients undergoing primary surgery. The Neonod2 study aims to include 850 patients, and the primary endpoint is 5-year Disease-Free Survival.

*In summary,* the data on de-escalation of axillary treatment are even more scarce in case of ypN+(SARP) disease than after ypN0(SARP) disease. The analysis of the national cancer database [63] suggests that replacing ALND by RNI yields a worse overall survival, in patients with cT1-3N+, ypN+(SLNB) disease. Preliminary results of the Dutch approach however, suggest that when replacing ALND with RT of level 1–4 is limited to patients with a low nodal burden prior to PST (i.e. ≤3 suspicious nodes at imaging), and ypN1(SARP) disease, it is oncologically safe (Table 3). However, again the results of several randomized trials are to be awaited before definitive conclusions can be drawn. If de-escalation of axillary treatment is considered such as in the Dutch approach, it is essential to at least register the outcome to allow evaluation of this progressive approach.

## 4 Further considerations

The above-mentioned findings and the cautious conclusions are in line with the conclusions drawn by Dubsky et al. [65] from a consensus meeting amongst European breast cancer experts. They also reached consensus (86%) that not all types of lymph node involvement shown at diagnosis necessitate ALND after PST. Especially in patients with limited nodal involvement prior to PST and a nodal pCR based on a negative SLNB and/or removal of a marked node by experienced surgeons, the panellists agreed that ALND is not indicated, recognizing that there are not many prospective trial data. They also agreed (93%) that not all these patients need RNI. The majority agreed that the indication for radiation volumes (64%) and the extent of volumes of the axilla (68%) depend on the presence of other risk factors as well. They further remarked that in spite of the limited available data replacing ALND with ART is increasingly supported.

Nevertheless, all authors justifiably warn that the above-mentioned data do not comprise level 1 evidence, and that the ongoing RCTs will have to be awaited before firm conclusions can be drawn. From a recent survey by the European Breast Cancer Research Association of Surgical Trialists (EUBREAST) it is clear that this lack of level 1 evidence leads to a wide heterogeneity in surgical approaches to the axilla after PST [66]. To overcome the current problem that in many (surgical) studies important radiation details are lacking, the Breast Cancer subcommittee of ESTRO guidelines is currently developing Quality Assurance guidelines regarding the reporting of radiation details in trials.

As is clear from the previous, tumour stage, patient's age and comorbidities also have to be taken into consideration for the individualization on axillary treatment after PST. For further improvements in individualization of RNI, studies will have to consider tumour biology in much more detail. For instance, several retrospective studies have found that triple negative disease remains an independent negative prognostic factor, even in case of pCR [42,43,[67], [68], [69]]. Other studies showed that the percentage of Tumour Infiltrating Lymphocytes (TILs) is closely correlated with pCR, nodal response and disease-free survival, in patients with cN + disease treated with PST, where a higher percentage of TILs is associated with improved outcome [[70], [71], [72]]. Other fast growing research areas which may help to individualize RNI are radiomics [73] and liquid biopsies [74]. In the future, linking radiomics or liquid biopsy-derived biomarkers to disease stratification, prognosis and therapeutic response could provide valuable information for personalized therapy. Finally, most of the studies on PST concern primary chemotherapy, and not primary endocrine treatment. The current guidelines of axillary surgery following PST do not discriminate between primary endocrine treatment or chemotherapy [75]. It should be realized that primary endocrine treatment is usually reserved for patients with biologically more favourable disease. After primary endocrine treatment patients may have a higher nodal response rate than after primary chemotherapy [76]. This may also have consequences for de-escalation strategies. Further studies are required into de-escalation strategies in these more favourable subtypes, who have been treated with primary endocrine treatment [75,76].

## 5 Conclusions

This review showed that there is accumulating evidence that de-escalation of RNI in patients treated with PST can be oncologically safe, if patients are adequately selected. Most studies select patients based on the 1) cN-status: either cN0 or cN+, where some studies make a further subdivision into Low Nodal Tumour Burden (≤3 suspicious nodes at imaging) and High Nodal Tumour Burden (>3 suspicious nodes at imaging), and 2) on the ypN status, using different surgical axillary restaging procedures (SARP), i.e. SLNB and/or the removal of a marked node (Table 2). Although level 1 evidence is still lacking, ALND is increasingly omitted or replaced with RNI in patients with cN + disease, who have undergone treatment with PST. The results of the currently ongoing RCTs have to be awaited before definitive conclusions can be drawn. We strongly recommend to apply de-escalation in prospective registration studies, including detailed recording of irradiated volumes, preferably according to the ESTRO guidelines that are currently being developed. Future studies should take into account biological subtypes, genetic profiles if available, and other biomarkers, to allow further individualization of regional nodal irradiation.

## Declaration of competing interest

None of the authors has expressed any conflict of interest, apart from being co-author of one or more of the reviewed papers.
